# When to anatomically excise a benign lung tumor-correspondence regarding the article: “large mesenchymal cystic and chondroid pulmonary hamartoma mimicking lung cancer: case report”

**DOI:** 10.1186/s13019-024-02554-9

**Published:** 2024-02-09

**Authors:** Fani Tsolaki, Georgios I. Tagarakis, Ioannis Tagarakis

**Affiliations:** 1 Aristotle University of Thessaloniki, University Campus, Thessaloniki, 54124 Greece; 2 Department of Cardiothoracic Surgery, Aristotle University of Thessaloniki, AHEPA University Hospital, Thessaloniki, 54636 Greece

**Keywords:** Lung tumor, Benign, Anatomic resection

## Abstract

The decision of whether to perform a large anatomic resection for a lung mass that is not definitely malignant comes often forward in the everyday practice of the thoracic surgeon. The general characteristics of the tumor as well as of the patient and the instinct and experience of the surgeon are the ones that dictate the final choice. Such a decision was made in the case of a large pulmonary hamartoma where a right middle lobectomy was performed with the postoperative course justifying the surgeons’ choice.

Dear Editor,

We read with interest the paper by Ahn et al. [1], published recently in your esteemed journal. The question of whether to perform a large anatomic resection or not in case of a sizeable lung tumor without a definite diagnosis of malignancy often comes forward in the everyday practice of a thoracic surgeon, sometimes even during the surgical procedure, when the pathologist cannot provide a clear answer based on the rapid biopsy specimen. Apart from the cases of doubt in regard to malignancy, lung excision for benign lesions can occur in various cases, such as tubercular lesions, complicated lung abscesses, symptomatic cavitary lesions caused by aspergillus, hydatid cysts or pulmonary sequestrations. In the described case, absence of smoking history and low SUV uptake in the PET-CT scan examination spoke in favor of a benign lesion, however the size of the mass and the age of the patient were signs for a possible malignancy. In such cases of doubt, the instinct and experience of the surgeon and the proper preoperative discussion and informed consent with the patient and relatives allowing multiple treatment options can provide the solution. The extent of the excision will be decided based on the experience of the surgeon (as sublobar anatomic excisions may be more technically demanding than a lobectomy) and the actual contribution of the affected lobe to the total respiratory function of the patient which may be minimal when the pathological lesion has inflicted a major part of the lobe.

In the commented case, Ahn et al. correctly explained that the bizarre appearance of the tumor with its size and cystic nature that rendered it prone to rupture and related complications (e.g. dissemination, pneumothorax), led them to the option of the right middle lobectomy, a decision with which we agree. The optimal postoperative course of the patient justified their choice.

## Data Availability

Not applicable.
